# Histone deacetylase enzymes as drug targets for the control of the sheep blowfly, *Lucilia cuprina*

**DOI:** 10.1016/j.ijpddr.2015.09.003

**Published:** 2015-10-09

**Authors:** Andrew C. Kotze, Barney M. Hines, Neil H. Bagnall, Clare A. Anstead, Praveer Gupta, Robert C. Reid, Angela P. Ruffell, David P. Fairlie

**Affiliations:** a CSIRO Agriculture, St. Lucia, Queensland 4067, Australia; b Faculty of Veterinary and Agricultural Sciences, The University of Melbourne, Parkville, Victoria, Australia; c Division of Chemistry and Structural Biology, Institute for Molecular Bioscience, The University of Queensland, St. Lucia, Queensland 4072, Australia

**Keywords:** *Lucilia cuprina*, Histone deacetylase, Insecticide, Trichostatin

## Abstract

The Australian sheep blowfly, *Lucilia cuprina*, is an ecto-parasite that causes significant economic losses in the sheep industry. Emerging resistance to insecticides used to protect sheep from this parasite is driving the search for new drugs that act via different mechanisms. Inhibitors of histone deacetylases (HDACs), enzymes essential for regulating eukaryotic gene transcription, are prospective new insecticides based on their capacity to kill human parasites. The blowfly genome was found here to contain five HDAC genes corresponding to human HDACs 1, 3, 4, 6 and 11. The catalytic domains of blowfly HDACs 1 and 3 have high sequence identity with corresponding human and other Dipteran insect HDACs (*Musca domestica* and *Drosophila melanogaster*). On the other hand, HDACs 4, 6 and 11 from the blowfly and the other Dipteran species showed up to 53% difference in catalytic domain amino acids from corresponding human sequences, suggesting the possibility of developing HDAC inhibitors specific for insects as desired for a commercial insecticide. Differences in transcription patterns for different blowfly HDACs through the life cycle, and between the sexes of adult flies, suggest different functions in regulating gene transcription within this organism and possibly different vulnerabilities. Data that supports HDACs as possible new insecticide targets is the finding that trichostatin A and suberoylanilide hydroxamic acid retarded growth of early instar blowfly larvae *in vitro*, and reduced the pupation rate. Trichostatin A was 8-fold less potent than the commercial insecticide cyromazine in inhibiting larval growth. Our results support further development of inhibitors of blowfly HDACs with selectivity over human and other mammalian HDACs as a new class of prospective insecticides for sheep blowfly.

## Highlights

• *Lucilia cuprina* genome contains five histone deacetylase genes.
• Significant amino acid differences between insect and human HDACs 4,6 and 11.
• Trichostatin highly toxic towards blowfly larvae.
• Potential for HDAC inhibitors as insecticides.

## Graphical abstract

**Figure:**
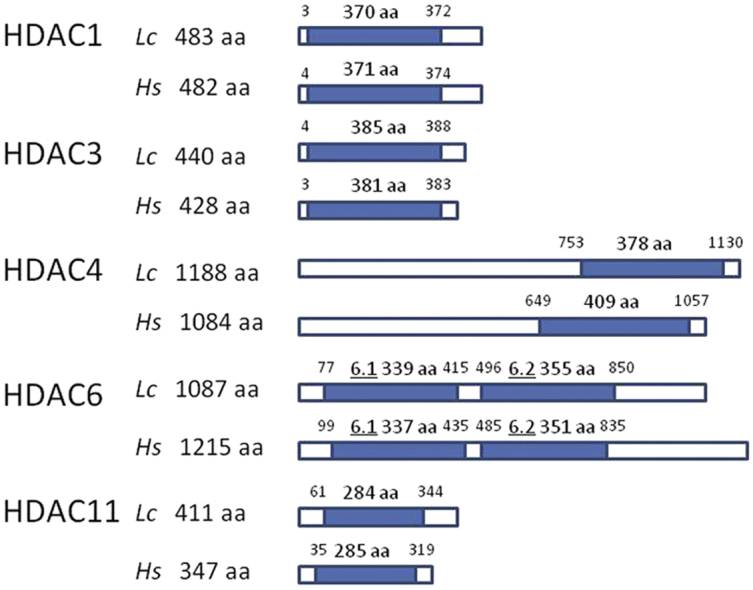

## 1 Introduction

The Australian sheep blowfly, *Lucilia cuprina*, is an important parasite of sheep in Australia. Adult *L. cuprina* females are attracted to sheep odours, particularly those associated with bacterial infections in damp fleece, or areas of fleece or skin soiled by urine or faeces (Watts et al., 1981, Emmens and Murray, 1983). In these regions of high humidity, each female fly lays approximately 200 eggs per batch. The eggs hatch and develop into larvae that abrade the skin with their mouth hooks, and secrete digestive proteases, to initiate the blowfly strike lesion (Sandeman et al., 1987, Sandeman et al., 1990). The larvae feed for several days, causing severe tissue damage, toxaemia and, in some cases, mortality. Blowfly strike is responsible for production losses in the Australian sheep industry amounting to over $AUD280M per annum (Sackett et al., 2006). The control of the parasite relies largely on the use of insecticides applied to the sheep as preventative treatments. These chemicals remain active against larvae hatching from freshly-laid eggs on the sheep for several months. However, the blowfly has now developed resistance to some of these insecticides, with organophosphate- and benzoylphenylurea-based products no longer being effective (Sandeman et al., 2014). Resistance to the widely-used substituted diaminotriazine compound cyromazine has also been reported recently (Levot, 2012, Levot et al., 2014). As the Australian sheep industry currently relies on such insecticides derived from this single chemical family to which resistance has been detected, there is an urgent need for new insecticides that preferably have a different mechanism of action.

Histone deacetylase (HDAC) enzymes are essential regulators of gene transcription in all eukaryotic organisms, catalysing removal of acetyl groups from lysine sidechains of nucleosomal histone proteins (Kou and Allis, 1998). They act in conjunction with histone acetyltransferases (which catalyse the reverse reaction) to control the degree to which histones are acetylated, and hence gene transcription patterns in cells. HDACs have been recognised as therapeutic targets in cancer for many years (Cairns, 2001), with more than ten HDAC inhibitors currently in use in the clinic or in clinical trials as anti-cancer drugs. HDAC inhibitors are also being studied for inhibition of human parasitic infections, including malaria, toxoplasmosis, trypanosomiasis, schistosomiasis and leishamaniasis (Andrews et al., 2009, Andrews et al., 2012). In insects the effects of HDAC inhibitors have been reported on gene transcription patterns in *Drosophila melanogaster* and on the activity of individual recombinant HDAC enzymes (Foglietti et al., 2006, Cho et al., 2005). There has been a single report examining the lethality for an HDAC inhibitor (trichostatin A) on *D. melanogaster* (Pile et al., 2001). The compound inhibited adult eclosion following exposure to embryos, and larvae failed to fully develop. We were therefore interested in the potential of HDAC inhibitors to be a new class of insecticides.

This study aimed firstly to identify all HDAC genes present in *L*. *cuprina*, taking advantage of the recently completed blowfly genome (Anstead et al., 2015), and to compare the blowfly HDAC genes with those of other insects and mammals. This information may also inform other prospective uses of HDAC inhibitors more broadly as insecticides other than for the control of the sheep blowfly alone. Knowledge on differences between insect and mammalian forms is crucial in order to ensure specificity and safety of HDAC inhibitors applied to sheep for blowfly control, or to other mammals as insecticides. We examined HDAC gene expression patterns through the life cycle of the blowfly in order to identify the best HDAC enzymes to target, and when the insect is potentially most vulnerable. Chemotherapeutic approaches to the control of the sheep blowfly are best directed at the larval life stages present on sheep, as it is not practical to target adult flies due to their mobility. Hence, information provided here on the expression of HDAC genes in early larval life stages is important in assessing their value as new drug targets for blowfly control. Finally, to establish proof of concept, two compounds that inhibit most HDAC enzymes were examined for toxicity against blowfly larvae using an *in vitro* bioassay, and compared to commercial blowfly insecticides. For this exercise, we chose to examine trichostatin A (TSA) because it is a well-known and potent pan-HDAC inhibitor (Yoshida et al., 1995) used widely to study HDAC inhibition *in vitro*. Suberoylanilide hydroxamic acid (SAHA, vorinostat) was also examined as this is perhaps the most common HDAC inhibitor used *in vivo* and in clinical studies, and is also approved for human use to treat cutaneous lymphomas (Iwamoto et al., 2013).

## 2 Materials and methods

### 2.1 Insects and chemicals

The *L. cuprina* flies used in this study were from the laboratory reference drug-susceptible LS strain. This strain was derived from collections in the Australian Capital Territory, and has no history of exposure to insecticides. It has been maintained in the laboratory for >40 years. Adult flies were kept at 28 °C and 80% relative humidity with a daily photoperiod of LD 16: 8 h. Adults were maintained on a diet of sugar and water; larvae were raised on a wheatgerm culture medium as described by Tachibana and Numata (2001). Gravid females were allowed to oviposit onto bovine liver before eggs were transferred to the wheatgerm culture medium shortly afterwards.

Trichostatin A (TSA), tylosin solution (8 mg/mL) and dicyclanil were purchased from Sigma Chemical Co., diflubenzuron and cyromazine from ChemService, and suberoylanilide hydroxamic acid (SAHA, vorinostat) from Cayman Chemical Co.

### 2.2 *Lucilia cuprina* HDAC genes

The recently-completed *L. cuprina* genome (Anstead et al., 2015) was searched using sequences for human HDACs 1–11. Homologous sequences in *L. cuprina* (E-value cut-off: ≤ 10^−5^) were confirmed by comparisons to human, *D. melanogaster* and *Musca domestica* (housefly) sequences in the National Center for Biotechnology Information database. Molecular phylogenetic analysis was conducted in MEGA6 (Tamura et al., 2013). The maximum likelihood method was used to construct a phylogenetic tree for the catalytic domain amino acid sequences for five HDACs from each of the three Dipteran species, as well as human HDACs 1–11. We used the percent identity matrix created by Clustal2.1 to generate identity matrix tables to separately compare the catalytic domains of each of the blowfly HDACs with those of the other two Dipterans and humans.

### 2.3 Life stage transcription profiling

Blowflies were collected at various stages through the life cycle in order to examine HDAC transcription patterns. All samples were snap frozen immediately in liquid nitrogen, and stored at −80 °C. Larval life stages were cultured in 70 mL containers as described by Kotze et al. (2014). Eggs were harvested (Day 0) by placing a slice of liver into a cage of mature adult flies from approximately 1200 h–1400 h. The liver slice covered in eggs was then removed, and batches of approximately 25 mg were added to 2 mL screw top vials containing a mixture of 0.1, 1.0 and 2 mm zirconian/silica beads (Biospec Products) and snap frozen. The remaining eggs were retained on the liver, and placed at room temperature in the dark overnight. The next morning (Day 1), batches of 25 mg of freshly hatched larvae were collected and snap frozen. A number of cultures were established by placing 50 larvae into containers with four layers of Whatman number 1 filter paper (GH Healthcare) and 0.2 g of chopped cotton wool soaked with 2.5 mL of a nutrient medium consisting of 80 g/L yeast extract (Merck) and 1.6 mg/mL tylosin (Sigma) in lamb serum (Life Technologies) buffered with 35 mM KH_2_PO_4_.2H_2_0, pH7.5. The larvae were fed with 1 mL of nutrient medium on Day 1, and then 2 mL on each of Days 2 and 3. Larvae were sampled on Days 2 (n = 20 individuals), 3 (n = 4) and 4 (n = 4) from the pots and snap frozen. The stage of development of larvae on Days 2, 3 and 4 was determined by examination of the posterior spiracle openings in 4 individuals (1, 2 and 3 openings expected for each spiracle in 1st, 2nd and 3rd instars, respectively).

Late on Day 4, the containers were placed into large pots with a layer of sand at the base to serve as a medium for pupation, and returned to the incubator. Pupae were sampled on Days 7 and 11, and snap frozen (4 individual pupae per sample). On Day 11, the remaining pupae were separated from the sand on a sieve, and placed into cages containing water and sugar cubes, at 28 °C (80% relative humidity, photoperiod of LD 16: 8 h). On Day 15, adult flies were collected, anaesthetised using CO_2_, and 3 males and 3 females were recovered and snap frozen. A slice of liver was placed into the cage on Day 16 to provide a protein meal for the adult flies. Samples of male and female flies (n = 3) were again taken on Days 19 and 23. Three separate time course experiments were performed.

RNA was extracted using an RNeasy kit (Qiagen, Hilden, Germany), as per the manufacturer's instructions, with initial homogenisation by shaking on a Powerlyzer 24 (Mo Bio Laboratories). Following extraction, the samples were quantified using a Nanodrop and treated with TurboDnase (Ambion) to remove any genomic DNA. RNA quality was assessed using an Agilent Bioanalyser. cDNA synthesis was performed on extracted RNA using SuperScript III Reverse Transcriptase (Lifetechnologies), according to the manufacturer's instructions.

Quantitative PCR primers were designed for each of the blowfly HDAC genes using Primer 3 software (Koressaar and Remm, 2007, Untergrasser et al., 2012) (Supplementary Table 1). Five housekeeper genes (18S rRNA, 28S rRNA, β-tubulin, RPLPO and GST1) were used as references for the normalisation of data across the various time points (Bagnall and Kotze, 2010). A 7900HT thermocycler (Applied Biosystems) was used with the SYBR Green dye system (Applied Biosystems) and the following PCR cycling conditions: 50 °C for 2 min, 95 °C for 10 min, followed by 40 cycles 95 °C for 15 s, 60 °C for 1 min, 95 °C for 2 min, 60 °C for 15 s. PCRs were run in quadruplicate. Reaction efficiencies were determined by performing PCRs using a series of four, 5-fold cDNA dilutions. Standard curves for all primer pairs indicated an efficiency range between 86 and 99%. Melting curve analysis of each primer pair identified the qPCR products to be homogenous.

The data were analysed using REST 2009 software in a two-step process:

i) Initially, each life stage was compared separately to the Day 1 first instar larval stage as the control sample. In this way the transcription of each HDAC was expressed relative to the transcription level of that specific HDAC on Day 1. This provided information on relative levels of transcription at the different time points within a specific HDAC.
ii) Secondly, the relative transcription levels of each HDAC in the Day 1 time point were compared. This allowed for the transcription levels across the five HDAC genes to be expressed relative to HDAC1 on Day 1, and hence provided a means to derive relative transcription values across all genes and all time points using the data generated in part i) above; for instance, if HDAC3 was transcribed at 10-fold lower levels on Day 8 than on Day 1 (from part i)), and if HDAC3 was expressed at 5-fold lower levels than HDAC1 on Day 1 (from part ii)), then the expression levels after correction became HDAC1 Day1 = 1, HDAC3 Day1 = 0.2, HDAC3 Day 8 = 0.02. In this way all the data were corrected to show transcription relative to HDAC1 on Day 1 which was assigned a value of 1.

Three separate relative transcription values were derived for each HDAC at each time point using the quantitative PCR date from the three separate time course experiments. The data were analysed using ANOVA following log_10_ transformation. Significant differences between the different time points for each HDAC, as well as between HDACs at each time point, were identified using Tukey's multiple comparison test at *P* = 0.05.

### 2.4 Bioassays

The effect of the HDAC inhibitors TSA and SAHA on larval growth was assessed using a bioassay system in which larvae were allowed to develop on cotton wool impregnated with the compounds at various concentrations (modified slightly from Kotze et al., 2014). The assay was in the format described above for the culturing of larvae for HDAC transcription profiling except that the cotton wool in each assay container was impregnated with drug prior to the addition of 50 freshly-hatched larvae on Day 0. The potency of the two HDAC inhibitors was compared to three commercial blowfly-control chemicals, cyromazine, dicyclanil and diflubenzuron. TSA and SAHA were prepared as stock solutions at 1 mg/mL in ethanol, while diflubenzuron was prepared at 1 mg/mL in acetone, and cyromazine and dicyclanil at the same concentration in distilled water. These stock solutions were kept at −80 °C. Working solutions, consisting of 4-fold dilutions in the various solvents, were also stored at −80 °C. Aliquots of these solutions (4 mL) were added to the cotton wool in bioassay containers one day prior to the addition of larvae, and the solvents allowed to evaporate overnight in a fume hood. Control containers were prepared by addition of 4 mL ethanol to the cotton wool.

In order to calculate mean larval weight at the beginning of the drug exposure period, two groups of 100 larvae were collected, blotted dry on paper towel, weighed and discarded on Day 0. After 24 h (Day 1), 3 larvae were removed from each container, weighed, and discarded. The remaining larvae were fed with nutrient medium on days 2 and 3, and then placed into the larger pupation containers, as described above. On Day 10, the pupae were separated from the sand on a sieve, and the pupae were counted. Each compound was examined at either three of four 5-fold serially diluted concentrations. Each experiment consisted of a single container at each concentration of HDAC inhibitor or insecticide, alongside 4 control assays. Three separate experiments were performed.

The effect of the compounds on larval development was defined in two ways:

i) effect on larval weight gain over the first 24 h by expressing the total weight gain of the 3 larvae sampled on Day 1 as a percentage of the mean of the weight gain of the 3 larvae sampled from each of the 4 control containers (weight gain measured using the mean weight of larvae on Day 0 as an initial weight);
ii) effect on the pupation rate by expressing the number of pupae in each drug-treated container as a percentage of the mean number of pupae in the 4 control containers.
  The larval weight and pupation rate dose–response data were analysed with GraphPad Prism^®^ software using non-linear regression, with the ‘variable slope’ option selected, in order to calculate IC_50_ values (with 95% Confidence Intervals) representing the concentration of inhibitor required to reduce the larval weight gain or pupation rate to 50% of that measured in control (no drug) treatments.

## 3 Results

**Fig. 1:**
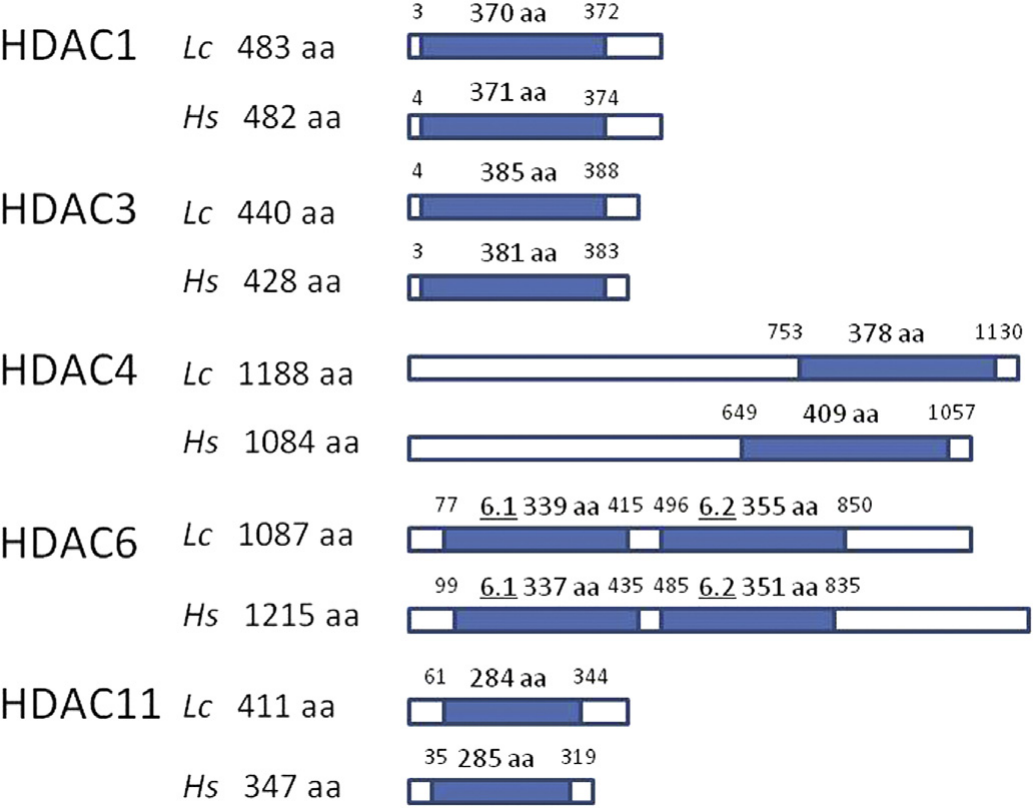
Schematic representation of the five blowfly (*Lc*) HDAC proteins, and their human (*Hs*) homologues, with the catalytic domains shaded. The amino acid lengths of the proteins are shown on the left, and the lengths of the catalytic domains are shown above each domain. The start and end amino acids for the catalytic domains are given at each end of the domains. The two catalytic domains of HDAC6 are shown as 6.1 and 6.2.

**Fig. 2:**
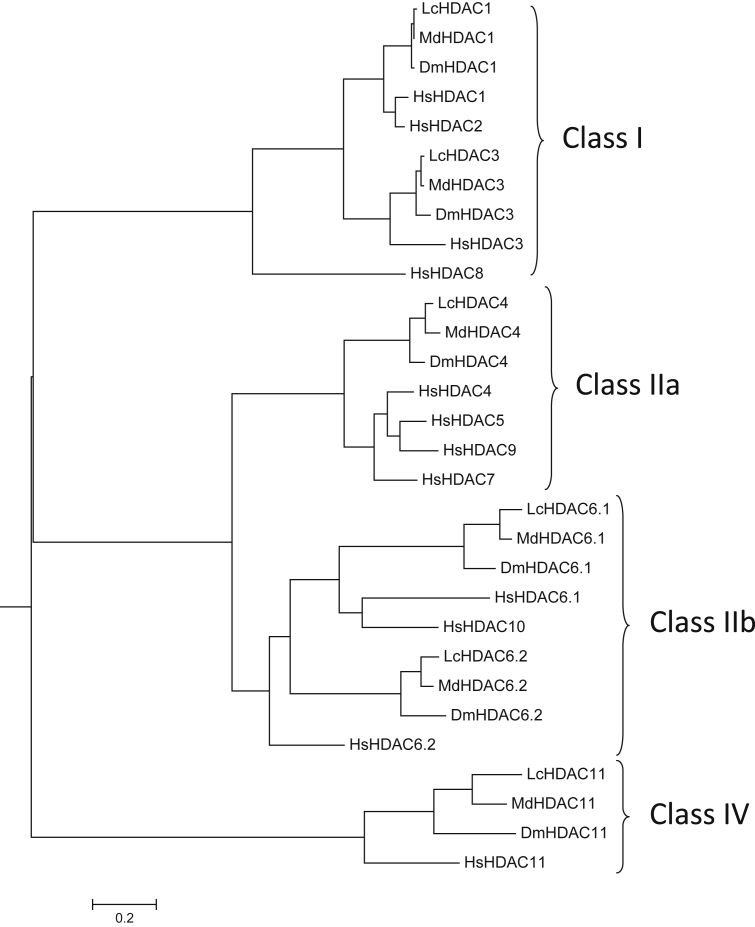
Phylogenetic tree illustrating relationships between the amino acid sequences of the catalytic domains of the eleven zinc-containing HDACs from three Dipteran species versus human (Lc *Lucilia cuprina*, Md *Musca domestica*, Dm *Drosophila melanogaster*, Hs *Homo sapiens*). The analysis was conducted in MEGA6 (Tamura et al., 2013). The tree is drawn to scale, with branch lengths measured as the number of substitutions per site.

Examination of the sheep blowfly genome revealed the presence of five HDAC genes with sequences corresponding to human HDAC1, HDAC3 (both Class I HDACs), HDAC4 (Class IIa), HDAC6 (Class IIb), and HDAC11 (Class IV). We therefore named the blowfly genes as follows: LcHDAC1 (GenBank accession no. **FF38_03544**), LcHDAC3 (**FF38_01208**), LcHDAC4 (**FF38_13781**), LcHDAC6 (**FF38_14519**) and LcHDAC11 (**FF38_06169**). Comparisons between the human and blowfly full-length amino acid sequences showed amino acid identities for HDAC1, 3, 4, 6 and 11 of 78%, 68%, 59%, 44% and 55%, respectively. The blowfly and human HDAC proteins are represented schematically in Fig. 1, with the catalytic domains highlighted. Both the full protein and catalytic domain lengths were similar for each blowfly and corresponding human HDAC. The phylogenetic relationships between the catalytic domains of the blowfly HDAC genes, those from two other Dipteran insects (*D. melanogaster* and *M. domestica*) and those from humans are shown in Fig. 2. The insect genes showed greater relatedness to each other than to the human genes in each case. Branch lengths separating the insect and human amino acid sequences were generally longer for the Class IIb and Class IV HDACs than for Class I and IIa.

**Fig. 3:**
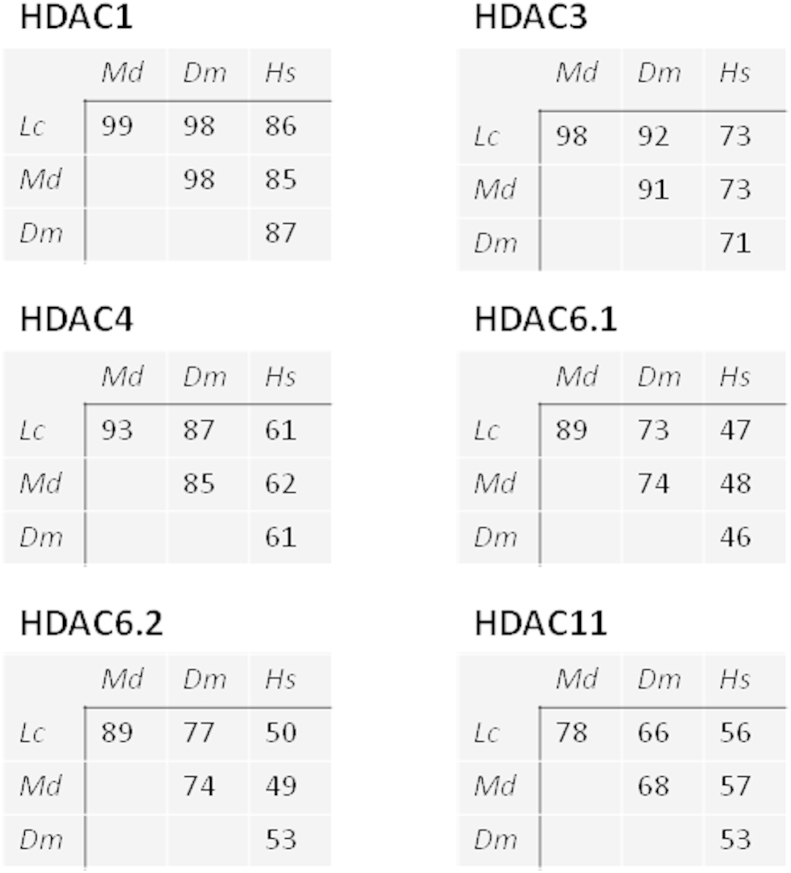
Comparison of HDAC catalytic domain amino acid residues between three Dipteran insects (Lc *Lucilia cuprina*, Md *Musca domestica*, Dm *Drosophila melanogaster*) and humans (Hs *Homo sapien*). Each separate figure component shows the percentage identity of amino acids in comparisons between pairs of species, for each HDAC gene. The values are taken from a percent identity matrix created by Clustal2.1.

The relatedness of the HDACs from the 4 organisms was examined further by comparing the percent identity of the catalytic domain amino acid residues for the three insects and humans (Fig. 3). The insect–insect comparisons showed greater degrees of identity than any insect-human comparison. Importantly, this analysis showed significant amino acid differences between insect and human HDAC proteins, particularly for HDACs 4, 6 (both 6.1 and 6.2) and HDAC11. The two catalytic domains of human HDAC6 showed only 46–53% amino acid identity to the equivalent domains in the three insects, while human HDAC4 and 11 showed 53–62% identity with the insects. HDAC 1 was the most similar in the three insects and humans.

**Fig. 4:**
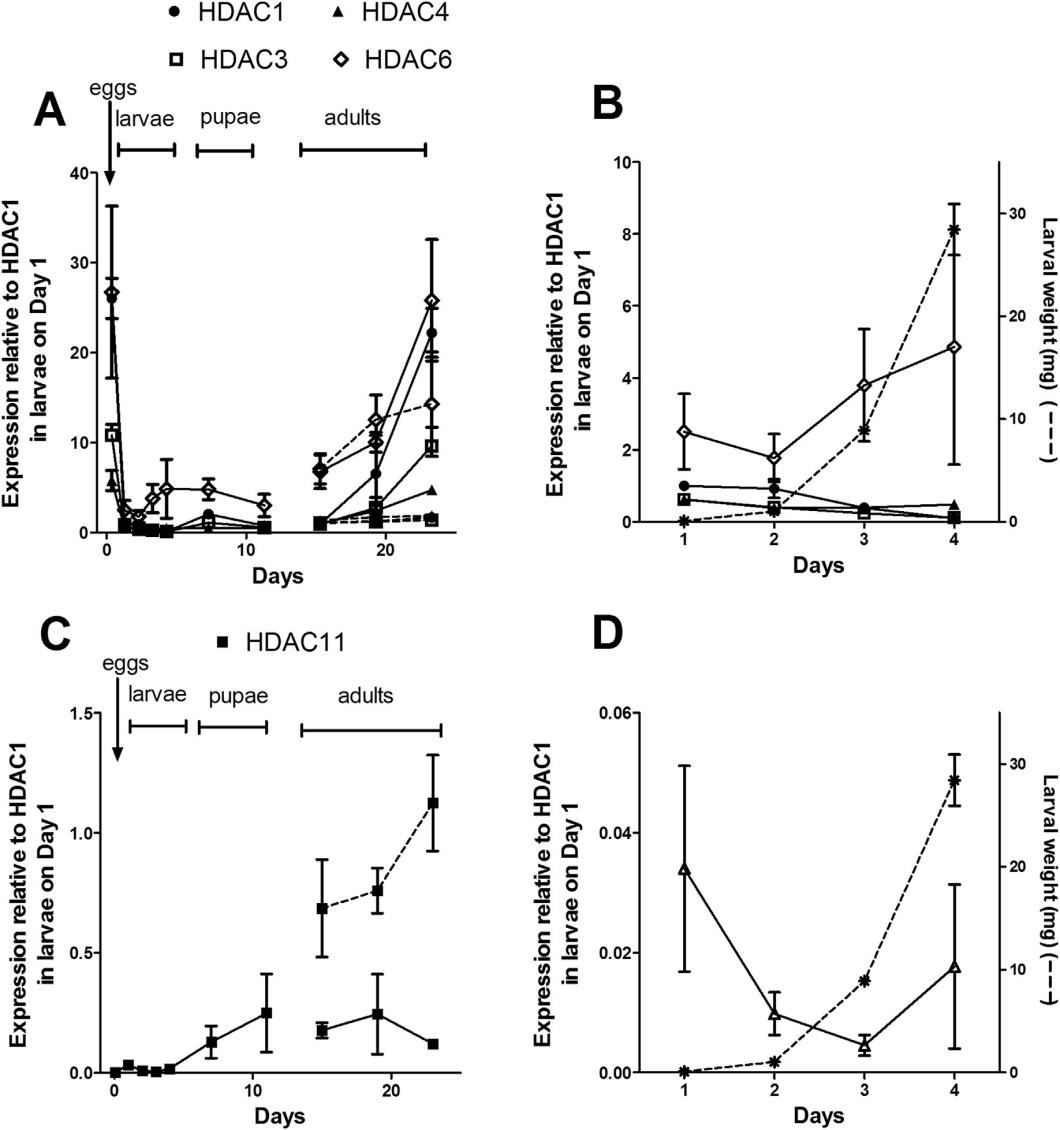
Transcriptomic profiling of HDAC genes across the blowfly life cycle. **A**: HDACs 1, 3, 4 and 6 across the whole life cycle; time points for larvae, pupae and adult females joined with solid lines, adult males with dashed lines; **B**: HDACs 1, 3, 4, and 6 in the larval stages only; larval weight shown as a dashed line; **C**: HDAC11 across the whole life cycle; time points for larvae, pupae and adult females joined with solid lines, adult males with dashed lines. **D**: HDAC11in larval stages only; larval weight shown as a dashed line. Each data point represents mean ± SE, n = 3 separate experiments. All data expressed relative to transcription of HDAC1 on Day 1 (freshly-hatched larvae), which was assigned a value of 1.

The transcription patterns for the five blowfly HDAC genes through the life cycle are shown in Fig. 4. The data were expressed with reference to the transcription level of HDAC1 at the second time point of the experiment (that is, at the freshly-hatched larval stage on day 1), which was assigned a value of 1. An examination of spiracle openings in larvae at each of the sampling time points across the larval phase of the experiment showed that the larvae were 1st instar on day 1, 2nd instar on day 2, and 3rd instar on days 3 and 4. The highest transcription of HDACs 1 and 3 occurred in the egg stage and in day 23 adult females, with transcription at these two stages being equivalent within each gene, at levels significantly higher than all the other life stages (Fig. 4A). Transcription of HDAC4 was higher in eggs than the other life stages, except for 19 day and 23 day females. The pattern was similar for HDAC6, with transcription in eggs higher than all the larval stages, alongside being equivalent to 19 and 23 day females and males. Within each HDAC, adult male flies showed equivalent transcription levels at each of the adult sampling times (days 15, 19 and 23). On the other hand, female flies showed significantly higher transcription levels at day 23 compared to day 15 for each of HDACs 1, 3, 4 and 6. Transcription patterns for HDACs 1, 3, 4 and 6 in the larval life stages alone, and the mean larval weight on each day, are shown in Fig. 4B. On days 1 and 2, HDAC6 was transcribed at equivalent levels to HDAC1, but was significantly higher than HDACs 3 and 4. On days 3 and 4, transcription of HDAC6 was higher than all the other genes.

Transcription levels were generally lower for HDAC 11 than for the other genes, especially in the larval stages, and hence, for clarity, it is presented separately (Fig. 4C and D). The transcription level was significantly lower throughout the larval stages than the level for HDAC1 on day 1 that was used as a reference point (with an assigned value of 1, as described above). HDAC11 did not exhibit the peak in the egg stage observed for HDACs 1, 3, 4 and 6 (Fig. 4C). Transcription remained at least 30-fold less than HDAC1 across the larval life stages (Fig. 4D). Adult males on days 19 and 23 showed higher levels of transcription than the larval stages, while female flies showed equivalent levels to larvae across the three adult time points.

**Fig. 5:**
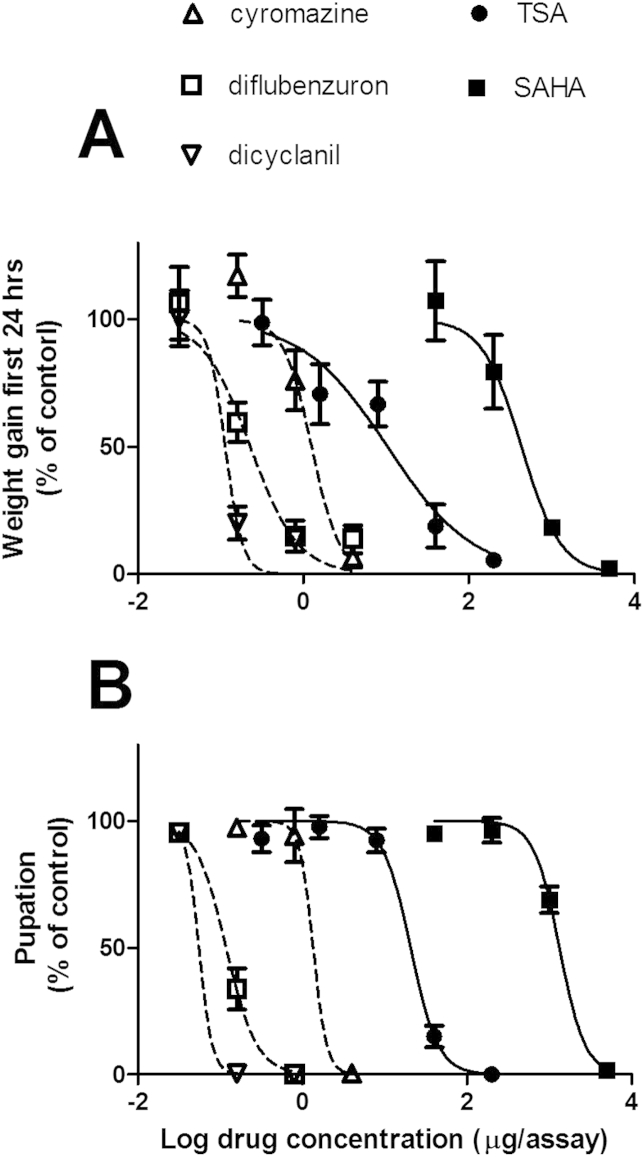
Effects of insecticides (dashed lines) and HDAC inhibitors (solid lines) on growth of blowfly larvae; **A**: effect on weight gain by larvae during the first 24 h of exposure to the compound; **B**: effect on pupation rate. Each data point represents mean ± SE, n = 3 separate experiments, each with a single assay at each compound concentration.

As proof of concept that HDAC enzymes might be useful targets for inhibition of blowfly growth, two pan-HDAC inhibitors were assessed alongside three commercial blowfly-control chemicals in *in vitro* bioassays for monitoring inhibition of growth of blowfly larvae. Dose-response curves (Fig. 5) compare the potencies of compounds in inhibiting growth of larvae during the first 24 h after egg hatch (Fig. 5A), as well as their effects on pupation rate (Fig. 5B). IC_50_ values are shown in Table 1. Both HDAC inhibitors showed dose-dependent inhibition of larval weight gain and pupation. TSA was more toxic than SAHA, 42- and 64-fold more potent in reducing weight and pupation, respectively. TSA was 8-and 90- fold less effective in inhibition of larval weight gain over the first 24 h than the commercial insecticides, cyromazine and dicyclanil, respectively. TSA and the three commercial insecticides each showed equivalent IC_50_ values for the weight gain and pupation measurements (overlapping 95% CIs for IC_50_s within each drug), while for SAHA the pupation IC_50_ was significantly greater than the weight gain IC_50_.

## 4 Discussion

A blowfly control agent must be directed at larval stages of blowflies feeding on sheep rather than the mobile adult stages that more widely inhabit the environment. A blowfly molecular target for an insecticide must therefore be present in larval stages, preferably in early larval stages (first and second instar), as they need to be killed before they extensively damage the host. Our transcription profiling has shown that the blowfly HDACs are generally transcribed at the highest levels in eggs and adult life stages, however each of the genes was also transcribed at readily detectable levels in the larval life stages. The peaks in egg and adult stages for HDACs 1, 3 and 4 are in general agreement with the patterns in *D. melanogaster* (Cho et al., 2005), whereas the peak in blowfly HDAC6 in eggs was not evident in *D. melanogaster.*

There was some commonality in the life-stage transcription patterns for the various blowfly HDACs, as well as key differences. For example, while transcription was generally highest in the eggs and in adult female flies for HDACs 1, 3, 4 and 6, HDAC11 did not show the peak in eggs. While transcription levels for HDACs 1, 3, 4 and 6 in adult males showed no change over the three adult sampling time points, levels of each gene increased significantly in females at day 23 compared to day 15. In addition, while HDACs 1 and 3 showed increases in adult females at day 23 compared to the larval stages, for HDAC11 only the adult males showed increases above larval levels. These different patterns most likely indicate different roles for the HDACs across the blowfly life cycle. Transcription profiling, RNA interference, and inhibitor studies with *D. melanogaster* have also suggested different roles for different HDACs in that fly, with evidence that individual HDACs regulate the transcription of distinct sets of genes (Cho et al., 2005, Foglietti et al., 2006).

This study has shown that the potent HDAC inhibitor TSA has significant toxicity towards the larval life stages of the sheep blowfly, thereby highlighting the potential of HDAC inhibitors to act as insecticides for sheep blowfly. Moreover, the potency of TSA was within 8-fold of a current blowfly control chemical, supporting the principle of inhibiting HDAC enzymes as a viable approach to a new kind of commercial insecticide. SAHA was significantly less toxic than TSA, consistent with similar differences between their relative potencies for killing cancer cells (Marks and Breslow, 2007) and parasites (Andrews et al., 2009) and inhibiting inflammatory diseases (Halili et al., 2009), and is simply due to differences in their relative cell permeabilities coupled with their relative affinities for HDAC enzymes inside cells (Gupta, 2012). The pupation IC_50_ for SAHA was greater than that for weight gain in the first 24 h, suggesting that the insecticidal effects of this compound decreased over the time course of the bioassay, and, hence, some larvae were able to recover to some extent from the inhibitory effects observed during the first 24 h of the assay. In contrast, the more potent TSA behaved in a similar manner to the commercial insecticides in showing equivalent IC_50_ values for the two measurements, simply reflecting its high potency.

A degree of insect specificity will be essential for any HDAC inhibitor to be used in the field as an insecticide. While TSA provides evidence for the lethality of a HDAC inhibitor to a Dipteran insect, this compound inhibits all HDAC enzymes and cannot itself be considered a viable insecticidal agent. The next goal will be to generate compounds that can selectively inhibit blowfly HDACs without affecting human, mammalian or other non-target insect HDACs. The selectivity of HDAC inhibitors as anti-parasitic agents more generally has only recently begun to be evaluated. There have been reports of selectivity of some HDAC inhibitors for malaria parasites versus mammalian cell lines (for example, Andrews et al., 2008, Dow et al., 2008, Wheatley et al., 2010), highlighting the potential to design inhibitors to selectively target non-mammalian HDAC enzymes. Our examination of HDAC catalytic domain amino acid sequences in the sheep blowfly and two other Dipteran insects, alongside the equivalent human proteins, provides some optimism that such insect specificity may be achievable. The catalytic domain amino acids of sheep blowfly HDACs 4, 6.1, 6.2 and 11 differed by up to 53% from the human homologs. Further analysis will be required to determine whether these amino acid differences affect the active site of the HDAC enzymes themselves, and hence are more likely to provide a degree of selectivity in the inhibitor actions, or whether they are at sites less important for inhibitor binding.

The comparison of amino acid sequences between the blowfly and human HDACs, alongside the transcription profiling data, highlights blowfly HDAC6 as potentially the most important insecticide target among the set of blowfly HDACs. The two catalytic domains of blowfly HDAC6 showed the least homology to their human homologues. HDAC6 was transcribed at higher levels than HDACs 3, 4 and 11, while being equivalent to HDAC1, at the day 1 time point of our sampling schedule, representing the early larval stages (1st instar) when it is most desirable to inhibit the development of the larvae before they damage the host.

Many HDAC inhibitors are unstable due to the hydroxamate component of their structures. While both TSA and SAHA are known to have short half lives in mammalian plasma at 37 °C (half life < 2 h) (Konsoula and Jung, 2008), both are typically used *in vitro* and in early *in vivo* studies to verify the importance of HDAC enzymes in biological systems. The stability of any proposed insecticidal HDAC inhibitor will be very important for any blowfly control chemical, and alternatives to hydroxamates as zinc-binding ligands in HDAC inhibitors are still under investigation. However, there are two aspects of inhibitor stability that are relevant: firstly, stability over long periods in the sheep fleece and on the skin surface (several months), and secondly, stability in protein exudates that form on the skin surface when larvae initiate a strike. The compounds will need to retain insecticidal activity in this protein exudate environment for a period of at least several days to ensure that the larvae derived from eggs laid by waves of gravid females over this period cannot develop beyond the early larval stage.

HDAC inhibitors have been studied with respect to their potential use as topical treatments, with a view to developing a means for rapid penetration into the skin for the treatment of melanoma and arthritis (for example, Chung et al., 2004, Gowda et al., 2012). On the other hand, as mentioned above, the use of HDAC inhibitors as blowfly control agents would rely on maintenance of drug within the skin surface/wool follicle environment for a period of several months, and hence would most-likely rely on the type of formulation chemistries currently used to bind blowfly control products to the lanolin on wool fibres (for example, Fleecebind™). A further consideration impacting on the potential use of HDAC inhibitors as insecticides for blowfly control will be the cost of such compounds. The costs able to be borne by livestock enterprises for parasite control are significantly less than those considered acceptable for drugs to be used in human health, particularly with respect to cancer therapeutics. Likely production costs will be an important component of future insecticide structure/activity studies and thus HDAC inhibitors with longer half-lives and greater stabilities should also not be too synthetically complex or expensive to produce.

In conclusion, the present study has identified important similarities and differences between HDAC sequences and their temporal expression in the sheep blowfly, *L*. *cuprina*. This information is valuable in assessing both the viability of each HDAC as a new insecticidal target, and the potential vulnerability of this species of fly to inhibition of different HDACs at different stages of its life cycle. The study has also provided early proof of concept that HDAC inhibitors might be viable as a new class of insecticides, with the activity of the pan-HDAC inhibitor TSA being within 8-fold of the *in vitro* potency of a commercial insecticide currently used to control sheep blowfly. Further work will require development of insect-specific HDAC inhibitors with sufficient potency and stability for use against insect pests in the field. High-throughput parallel assays using recombinant insect HDACs and mammalian enzymes may provide a means of identifying potent insect-specific compounds rather than relying on whole organism bioassays as demonstrated here. The sequences of blowfly and other insect HDACs studied here might also be aligned with crystal structures of human HDACs to construct three dimensional structural models of blowfly and other insect HDACs. Such structural models might provide valuable insights into structural requirements needed for developing HDAC-directed drugs with insect selectivity.

## Figures and Tables

**Table 1:** Response of *Lucilia cuprina* larvae to HDAC inhibitors and insecticides.

Compound	Weight gain in first 24 h		Pupation	
IC_50_ (μg/assay)	95% CI	IC_50_ (μg/assay)	95% CI
TSA	10.4	5.3–20.4	20.6	16.0–26.6
SAHA	434	247–763	1327	1026–1717
Cyromazine	1.27	0.62–2.59	1.36	0.05–34.48
Diflubenzuron	0.23	0.13–0.40	0.12	0.09–0.15
Dicyclanil	0.12	0.01–0.97	0.06	0.03–0.10
