# Supplementary material for: Histone deacetylase enzymes as drug targets for the control of the sheep blowfly, Lucilia cuprina
Source: Int J Parasitol Drugs Drug Resist. 2015 Oct 9;5(3):201–8. doi: 10.1016/j.ijpddr.2015.09.003 (PMC4847001; doi:10.1016/j.ijpddr.2015.09.003)
Supplement: Supplementary file 1 [file mmc1.docx]

Supplementary Table 1.

Primers used for quantitative PCR of blowfly HDACs and reference genes

| Gene | Primer sequence 5’ – 3’ |
| --- | --- |
| LcHDAC1 | F: AACTTTCCAACCAGCTGCTG  R: ACAAAATCCACACAGCGACC |
| LcHDAC3 | F: AGGTCATGGTGCCTGTGTAG  R: TTGCGTAAAGTGTAACCGCC |
| LcHDAC4 | F: ACAATCGTCCTCCACCATGT  R: GGGGTAAAGGAGCTGACTGT |
| LcHDAC6 | F: TCAATGCCCTGCCATGAATG  R: CGCAAGGATAACACCAGACG |
| LcHDAC11 | F: AGAGGGTGTCATCGAACGAG  R: CAGACGCCTTCAAATAGCCG |
|  |  |
| 18S rRNA^a^ | F: AGCAGTTTGGGGGCATTAG  R: GCTGGCATCGTTTATGGTTAG |
| 28S rRNA^a^ | F: CCAAAGAGTCGTGTTGCTTG  R: ATTCAGGTTCATCGGGCTTA |
| β-tubulin^a^ | F: AAGCTGACGACACCCACATAC  R: CGGGCATGAAGAAGTGAAGA |
| RPLPO^a^ | F: ACCCATAAGGACGACACC  R: GGTGCTGACAATGTTGGTTC |
| GST1^a^ | F: GCCAGTGTCAGCACCTTTG  R: GCAACCTTCCCAGTTTTCATC |

^a^ reference gene primer sequences taken from Bagnall and Kotze (2010)

Bagnall NH, Kotze AC. 2010. Evaluation of reference genes for real-time PCR quantification of gene expression in the Australian sheep blowfly, *Lucilia cuprina*. Med Vet Entomol. 24:176-81.
